# Expanding the Reactive Sulfur Metabolome: Intracellular and Efflux Measurements of Small Oxoacids of Sulfur (SOS) and H_2_S in Human Primary Vascular Cell Culture

**DOI:** 10.3390/molecules26237160

**Published:** 2021-11-26

**Authors:** Ottis Scrivner, Ahmed Ismaeel, Murugaeson R. Kumar, Kristina Sorokolet, Panagiotis Koutakis, Patrick J. Farmer

**Affiliations:** 1 Department of Chemistry and Biochemistry, Baylor University, Waco, TX 76798, USA; ottis_scrivner@baylor.edu (O.S.); mraveekumar@gmail.com (M.R.K.); kristina_Sorokolet@baylor.edu (K.S.); 2 Department of Biology, Baylor University, Waco, TX 76798, USA; Ahmed_Ismaeel@baylor.edu (A.I.); Panagiotis_Koutakis@baylor.edu (P.K.)

**Keywords:** hydrogen sulfide, SOS (small oxoacids of sulfur), primary vascular cells, hypoxia

## Abstract

Hydrogen sulfide (H_2_S) is an endogenous signaling molecule which is important for cardiovascular health, but its mechanism of action remains poorly understood. Here, we report measurements of H_2_S as well as its oxidized metabolites, termed small oxoacids of sulfur (SOS = HSOH and HOSOH), in four human primary vascular cell lines: smooth muscle and endothelial cells derived from both human arterial and coronary tissues. We use a methodology that targets small molecular weight sulfur species; mass spectrometric analysis allows for species quantification to report cellular concentrations based on an H_2_S calibration curve. The production of H_2_S and SOS is orders of magnitude higher in smooth muscle (nanomolar) as compared to endothelial cell lines (picomolar). In all the primary lines measured, the distributions of these three species were HOSOH >H_2_S > HSOH, with much higher SOS than seen previously in non-vascular cell lines. H_2_S and SOS were effluxed from smooth muscle cells in higher concentrations than endothelial cells. Aortic smooth muscle cells were used to examine changes under hypoxic growth conditions. Hypoxia caused notable increases in HSOH and ROS, which we attribute to enhanced sulfide quinone oxidase activity that results in reverse electron transport.

## 1. Introduction

Like nitric oxide (NO) and carbon monoxide (CO), hydrogen sulfide (H_2_S) is a gasotransmitter that has been shown to be involved in many regulatory, cytoprotective, and signaling roles in human health, especially in cardiovascular diseases [1,2,3,4,5,6]. Several enzymes have been identified as physiological sources of endogenous H_2_S. In the cytosol, cystathionine-γ-lyase (CSE) and cystathionine-β-synthase (CBS) convert L-cysteine to yield H_2_S [7,8]. In mammals, CBS is important in producing H_2_S in the central nervous system [9], whereas CSE is thought to dominate H_2_S production in the cardiovasculature [10]; both enzymes are found concurrently expressed in many other tissues, e.g., the liver and kidney [11,12]. In the mitochondria, 3-mercaptopyruvate sulfotransferase (3MPST) utilizes L-cysteine aminotransferase (CAT) to produce H_2_S from the substrate 3-mercaptopyruvate [13] and is found throughout the body [14].

In the vasculature, the activity of H_2_S and NO are entwined [15]. It has been suggested H_2_S is predominately generated in vascular smooth muscle cells and nitric oxide in endothelial cells [16,17]. In mice, the endogenous concentration in aortic tissue was 20 to 100 times greater than that observed for the other tissues such as brain, liver, and heart [18]. Plasma-free H_2_S levels are significantly elevated in patients of cardiovascular disease [19], and decreased plasma H_2_S levels correlate with the severity of the disease and changes of the coronary artery [20]. Importantly, exogenous H_2_S shows relatively low activity in physiological assays, e.g., effecting vasodilation at non-physiological concentrations from 50 to 100 μM in vivo [16,21], and above 100 μM in vitro [22,23]. Thus, there is speculation that vasorelaxation and other cardiovascular effects are induced through biotransformation resulting in persulfide (e.g., RSSH) [24] or other modifications of protein thiols that can alter biochemical activity [25]. However, the mechanisms of such S-S catenations, as well as the selectivity, specificity, and control of targeted proteins are ill-defined [26]. Two biosynthetic pathways to persulfidation have been identified, but which do not depend on exogenous H_2_S levels; cysteinyl-tRNA synthetases generate cysteine polysulfides (CysSS_n_H) during translation [27,28] and 3-mercaptopyruvate sulfurtransferase and cysteine aminotransferase produce both H_2_S and persulfides H_2_S_n_ (*n* = 1–3) as well as cysteine- and glutathione-persulfides [29].

The endogenous levels of H_2_S in cells and tissues have been extensively studied, but there is still little consensus over its quantification [30], with reported levels in the pM to low nM range [31]. As H_2_S is easily oxidized and exists as a 75/24/1 mixture of H_2_S/HS^−^/S^2−^ congeners at physiological pH of 7.4 [32], different probes will necessarily possess different reactivities depending on the technique used. The earliest approaches involved colorimetric and fluorescence methodologies [33,34,35,36]; more recently a common method of detection was by trapping in situ with monobromobimane, MBB, which allowed characterization by both fluorescent measurement and mass spectroscopy [37]. Recent work, however, has found that the high concentrations of MBB needed for efficient trapping also induce cleavage of polysulfide bonds and can alter the sulfide/sulfane ratio [38]. Several new alkylators, such as HPE-IAM, β-(4-hydroxyphenyl)ethyl iodoacetamide (HPE), have been developed which minimize degradation and are highly efficient in mass spectroscopic characterizations [39,40]. Sulfur-oxide species such as cysteine sulfenic acids have been trapped by nucleophilic 5,5-dimethyl-1,3-cyclohexanedione (dimedone, DH), suggested to specifically target S-O bonds in cysteine sulfenic acids [41,42]. Dimedone trapped species may be cleaved with dithiothreitol and attributed to per- or poly-sulfide oxides [43]. Recently, it was found that dimedone could also cleave mixed persulfides bound to the electrophilic alkylator 4-chloro-7-nitrobenzofurazan, which also blocks sulfenic acids, thus allowing distinction between these species [44].

**Scheme 1:**
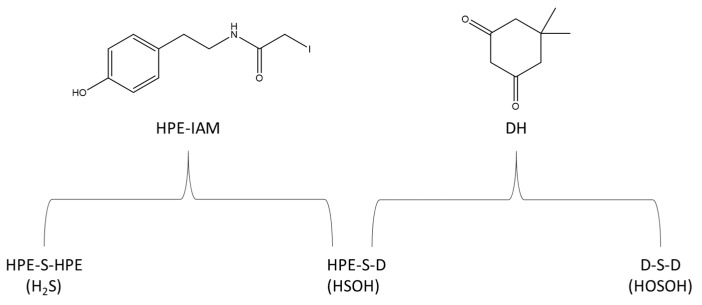
The use of traps S-H trap β-4-hydroxyphenyl ethyl iodoacetamide (HPE-IAM) and S-O trap dimedone (DH) to derivatize analytes into stable species observable by LC HD MS.

We and others have proposed that sulfhydration proceeds through the action of small oxoacids of sulfur (SOS) [45], simple metabolites of H_2_S such as sulfenic and sulfoxylic acids, HSOH and HOSOH, shown in Scheme 1, analogous to cysteine sulfenic and sulfinic acids [46,47]. Initially, we found that species such as HSOH, HOSOH, and other S-catenated species including polysulfides and polysulfide oxides such as thiosulfenic acids were generated by the oxidation of H_2_S with a variety of different oxidants such as peroxides, metmyoglobin and nitroso-glutathione, all of which can be found endogenously in biological systems [48,49]. These species were observed trapped in situ using the nucleophilic dimedone, DH, and an electrophilic trap iodoacetamide IAM [50], and then analyzed by liquid chromatography separation and high-definition mass spectrometry, LC HD MS.

The sulfur oxoacids are reactive and transient species; thus, it has been suggested the trapped SOS originate from the hydrolysis or decomposition of polysulfides [45]. Other studies have shown that per- and polysulfide/sulfane (RSSH/RSSSR) species interconvert and change over time [51,52,53]. Thus, the trapped SOS reflect a broad measure of S-oxidation level within as system, perhaps an indicator of sulfur metabolism if measured in cells or tissues.

With this intent, we translated the LC HD MS method to observe and quantify levels of trappable SOS and H_2_S in a panel of human cancer and non-cancer cell lines, bacteria, and yeast [54]. The new method involved precipitating and filtering the protein from cell lysates, to select for only small molecule analytes. We used Akaike’s more efficient β-(4-hydroxyphenyl)ethyl iodoacetamide (HPE-IAM) in place of iodoacetamide [39], measuring H_2_S as HPE-S-HPE, HSOH as HPE-S D, and HOSOH as D-S-D as shown in Scheme 1. The concentrations of traps were adjusted to maximize the yields seen while also minimizing cellular toxicity. The samples were analyzed in a similar methodology as described previously using LC HR MS to generate single-ion chromatograms, which were then converted to concentrations using a calibration curve of HPE-S-HPE created from derivatizing Na_2_S with HPE-IAM; thus, the concentrations reported here assume of equivalent efficiencies for all the analytes. Likewise, cell counting and estimated average cell volumes were used to calculate the concentrations of H_2_S and SOS.

In a previous report, the analyte concentrations varied dramatically between cell lines, from picomolar to nanomolar, and were affected by small molecule inhibitors of the H_2_S generating enzymes CSE, CBS, and CAT. As oxidized sulfur species are reactive and known to interconvert [55], we proposed the sulfomic index defined the percentage of observed electrophilic S bonds, i.e., the percentage of dimedone traps within all the trapped analyte species. An important observation was that these small analytes were extruded from cells at higher relative concentrations than within the cells, verified by washing cells with buffer, and trapping the extruded analytes within the separated wash solutions.

This report extends the method to characterize H_2_S and SOS concentrations in primary human vascular endothelial and smooth muscle cells, ECs and SMCs, and examine how hypoxia affects the observed concentrations. Although there is a precedent for SMCs having higher concentrations of H_2_S than ECs, there is no consensus on physiological concentrations due to variations in methods used as well as differences in sample normalization (e.g., μg of protein, tissue sample, or number of cells per sample) [17]. This has resulted in a range of reported concentrations from pM in cells to μM in tissue or plasma.

In ECs, exogenous H_2_S has been shown to influence a number of important signaling pathways; vascular endothelial growth factor receptor (VEGFR)2, which controls proliferation and angiogenesis [56]; mitogen-activated protein kinase (MAPK), which effects protein kinase B (Akt), extracellular signal-regulated kinase (ERK), and p38 [57]; sirtuin (SIRT)1, which increases in capillary density [58]; and upregulation of hypoxia-inducible factor-1 alpha (HIF-1α) under hypoxic conditions [59]. The role of H_2_S in vasodilation as well as in EC stimulation of SMCs are the most widely studied [60,61,62,63,64,65,66,67,68].

In SMCs, H_2_S is considered an antiproliferative agent working against hypoxia-induced proliferation by upregulating cyclooxygenase (COX)-2/prostacyclin (PGI2) [69], reducing proliferative cell nuclear antigen (PCNA) and cyclin D1 expression [70], and decreasing the expression of α5-/β5-integrins and matrix metalloproteinase-2 [71]. H_2_S has also been shown to support pro-apoptotic upregulation of ERK/p38 as well as caspase-3 activation [72,73]. Similar to ECs, SMCs are affected by H_2_S-induced vasodilation through activation of ATP-sensitive (K_ATP_) and cell membrane hyperpolarization [74], increasing guanosine 3′,5′-cyclic monophosphate (cGMP) through phosphodiesterase inhibition [75], and a possible reactivity with nitric oxide (NO) resulting in lower NO bioavailability [76]. Thus, the role of H_2_S in controlling important vascular processes is well established, but how it may exert differential effects in ECs and SMCs remains unclear.

The effects of H_2_S on mitochondrial function also play a role in vascular response to hypoxia and reperfusion [77]. At high levels, H_2_S inhibits cytochrome c oxidase in complex IV of the electron transport chain, which leads to its known toxicity [78], but at low concentrations, H_2_S appears to increase mitochondrial efficiency [79] and oxygen consumption rate [80]. The mitochondrial enzyme sulfide, quinone oxidoreductase (SQOR), within the inner membrane oxidizes H_2_S to reduce coenzyme Q (CoQ) [81,82]. This is especially important as hypoxia-treated cells have been shown to have approximately threefold increases in SQOR expression [83]. Electron equivalents from reduced CoQ can either contribute to forward reductions of complexes III and IV, or be transferred back to oxidized NAD^+^ in complex I in a process known as reverse electron transport [84]. Notably, in HEK293 cells, SQOR oxidation of H_2_S has been shown to drive complex I reverse electron transport, increased ROS generation, and reduced oxygen consumption [85]. Additionally, persulfidation of proteins resulting from of SQOR may stimulate mitochondrial biogenesis, mitophagy, increased mitochondrial enzyme activity, and increased mitochondrial bioenergetics [78].

## 2. Results

### 2.1. Measurements of Intracellular Concentrations

**Scheme 2:**
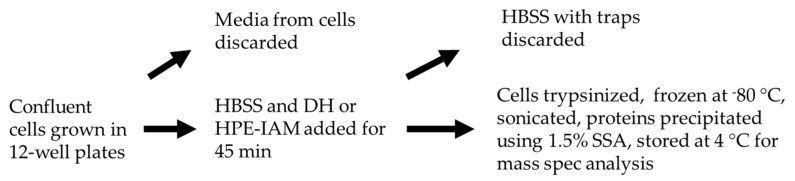
Flowchart showing methodology used to measure endogenous H_2_S and SOS in cell culture.

Scheme 2 shows the sample preparation sequences used to determine endogenous levels of H_2_S and SOS. Cells were grown to 70–80% confluency, the media was discarded, and cells were treated in three batches in HBSS buffer; one treated with HPE-IAM only, which was used to assess measure HPE-S-HPE; one treated with DH only to assess the D-S-D level; and one treated with the combination of HPE-IAM and DH to assess the HPE-S-D level. To minimize the effect of cell media on the measurements, the cells were washed, and the media was replaced with HBSS during trapping, as shown in Scheme 2, before being frozen prior to analyses. Each treatment was performed in triplicate over 45 min; the cells were washed, lifted, and frozen for storage. Subsequently, the cells were thawed and sonicated, proteins precipitated, and the filtrate stored prior to LC separation and analysis on an HD MS.

We then used selective ion chromatogram (SIC) peak areas of the derivatized analyte species to determine intracellular concentrations, as illustrated in Supplemental Figure S1. The SIC peak areas were first converted into analyte solution concentration using an H_2_S calibration curve, Figure S2, and then normalized to the number of cells per sample counted by hemocytometer using trypan blue staining. These numbers were then adjusted by calculating average cell volumes based on a hypothetical cell diameter as determined from literature and cell vendor information, Figures S3 and S4, to determine intracellular concentrations.

The results show much higher concentrations of all the analytes in SMCs, with aortic SMCs exhibiting higher amounts than coronary, as shown in Table 1. Total concentrations of small molecule S species, [H_2_S + SOS], are on the order of 4–10 micromolar in the SMCs, and from 0.2 to 0.4 micromolar in ECs. This straightforward comparison of SMCs to ECs across two different vascular tissue types concretely shows that both H_2_S and oxidized derivatives are 20–40× higher in SMCs than ECs in aortic and coronary cells.

**Figure 1:**
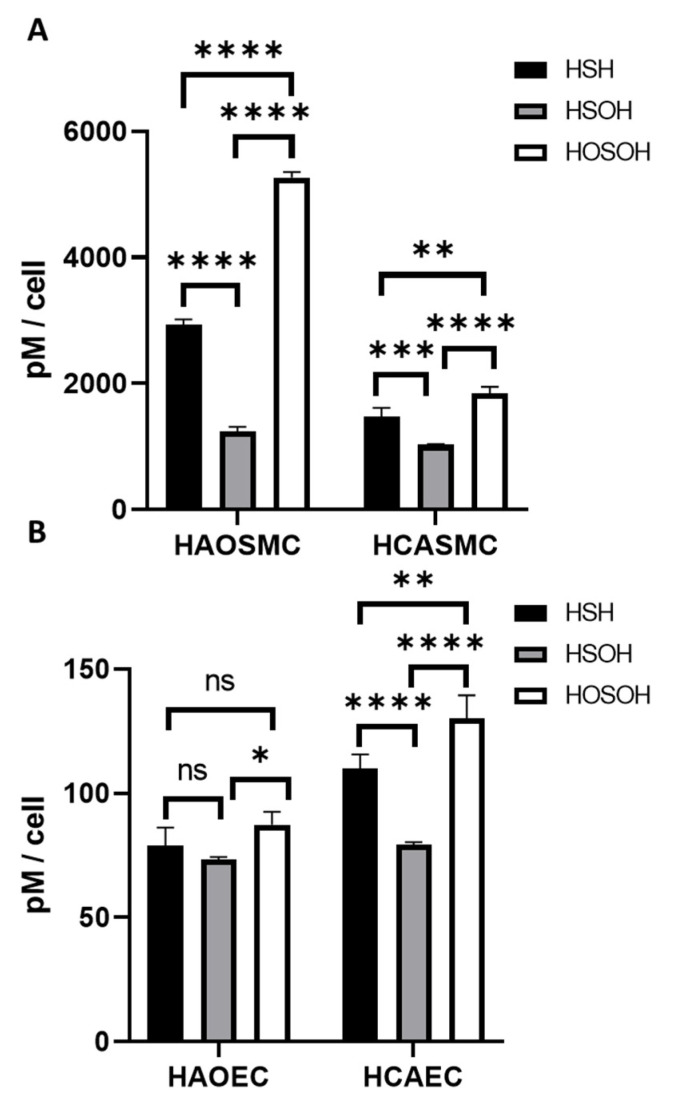
Intracellular endogenous concentrations of analytes in (**A**) human aortic/coronary smooth muscle cells and (**B**) human aortic/coronary endothelial cells. * = *p* < 0.05; ** = *p* < 0.01; *** = *p* < 0.001; **** = *p* < 0.0001; ns = not significant.

All four cell types displayed similar ranking of species, with HOSOH > H_2_S > HSOH, Figure 1; this ordering is distinctly different than previous measurements in noncancer and cancer lines in which H_2_S > HOSOH > HSOH, with concentrations of the oxidized species dramatically smaller than H_2_S. Table 2 shows normalized ratios of the analytes, which more clearly illustrates differences in S oxidation changes between different cell types. Far higher concentrations of SOS are seen in all four human primary vascular cell lines when comparing to previous data on HEK293, HeLa, and A375 cell lines [54]. The sulfomic index is also presented, a ratio of nucleophilic and electrophilic traps within the analytes, represented as the ratio of S-D concentration divided by the total S concentration, i.e., ([HSOH] + 2[HOSOH])/([H_2_S] + [HSOH] + [HOSOH]). This number ranges from zero to two and is representative of the relative oxidation state of small S-species in the sample. As seen in Table 2, the four primary vascular cell lines show high sulfomic indexes above 1, indicating that the majority of small molecule S-species are trapped by dimedone.

### 2.2. Measurements of Extracellular Concentrations

**Scheme 3:**
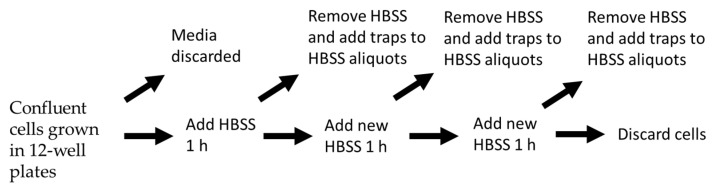
Flowchart showing methodology used to measure efflux of H_2_S and SOS species in cell culture.

A key finding in the previous study was that the S-analytes were extruded from the cells at effective concentrations higher than that within the cells. The same phenomena is seen for the primary vascular cells studied here. Scheme 3 illustrates the sample preparation for these measurements. The cells were sequentially washed with HBSS buffer, the wash transferred to a centrifuge tube for trapping, and three different batches of wash were treated with different trapping reagents as described for the endogenous measurements. The effluxed data are presented as rates ([H_2_S]/h) or ([SOS]/h) averaged over three separate re-equilibration of buffer events (see Figures S5 and S6 for each separate equilibration event for each time point).

**Figure 2:**
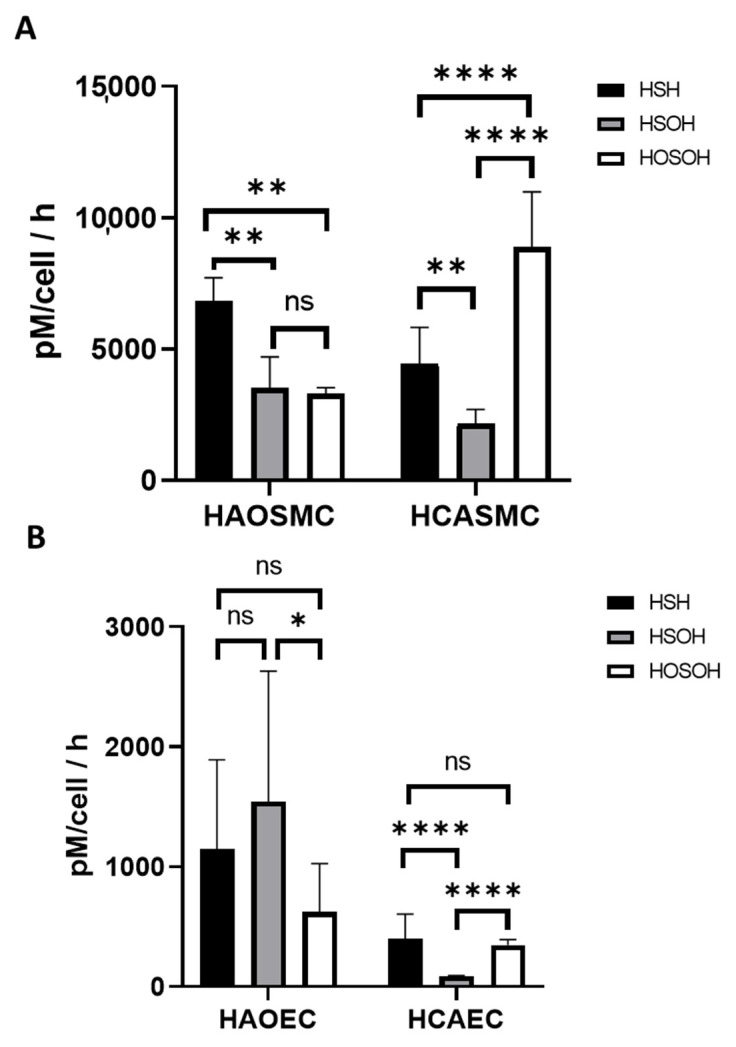
Rates of efflux of analytes averaged over three 1 h periods in (**A**) human aortic/coronary smooth muscle cells and (**B**) human aortic/coronary endothelial cells. * = *p* < 0.05; ** = *p* < 0.01; **** = *p* < 0.0001; ns = not significant.

The results show that small S-species generated within the cells are effluxed at high concentrations over time. Higher effluxes of both H_2_S and SOS are seen for SMCs compared to ECs, as shown in Figure 2 and Table 3. Again, the small S analytes appear to be extruded against a concentration gradient, as the efflux concentrations are 2–10 times higher than intracellular concentrations over similar trapping time scales.

Table 4 gives a comparison of efflux concentrations and sulfomic indexes by cell line, including data from previous study. Again, the concentration of S-analytes from smooth muscle cells is dramatically higher than all the others. Lower levels of S-oxidation are seen in the extruded compared to endogenous, as seen comparing sulfomic indexes in Table 2 and Table 4. However, when compared to the cancer and noncancer lines, these vascular primary cell lines do extrude significantly more oxidized species, as represented by higher sulfomic indexes.

### 2.3. Hypoxia Induced Changes of Endogenous and Effluxed Analytes in HAOSMCs

Much of the literature suggests that H_2_S plays a crucial role in response to hypoxia in both ischemia-reperfusion injuries and cardiovascular diseases [86,87,88]. Of particular significance is the apparent H_2_S moderation of SMCs proliferation under hypoxic conditions [89]. Therefore, HAOSMCs were chosen to assess the effects of hypoxia on the intracellular and extracellular concentrations of all the analytes in comparison with O_2_ consumption and ROS generation. These experiments were conducted in a HypoxyLab™ (Oxford Opronix Ltd. Milton Park, Abingdon, UK) bench-top incubator, which maintains the oxygen concentration at 1% O_2_ and allows the manipulations required to perform the trapping methodology. The cells were maintained under these conditions for 48 h prior to measurements, which previous literature suggests adequately represents a chronic hypoxia model [90].

**Figure 3:**
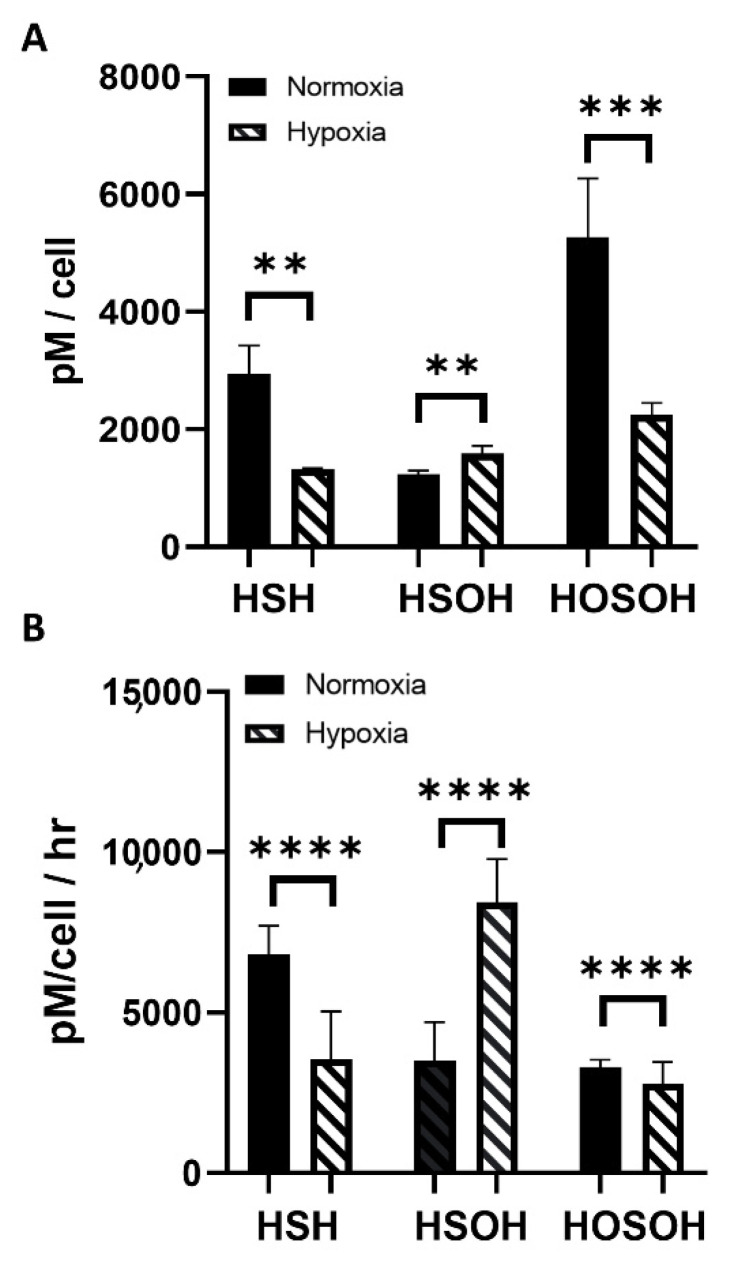
(**A**) Intracellular concentrations of analytes in HASMCs during normoxic and hypoxic growth and (**B**) rates of efflux of analytes averaged over three 1 h periods in HASMCs during normoxic and hypoxic growth. ** = *p* < 0.01; *** = *p* < 0.001; **** = *p* < 0.0001; ns = not significant.

As Figure 3 shows, growing HAOSMCs in hypoxic conditions led to significant changes in the measured analytes. Both intracellular and extracellular measurements followed similar trends: under hypoxia concentrations of H_2_S and HOSOH, they decrease, but HSOH increases, especially in the extracellular efflux (Figure S7). Under hypoxic conditions, intracellular H_2_S is the lowest in concentration, which suggests it is actively being consumed. Furthermore, as these species were trapped in a hypoxic incubator, the higher concentrations of extracellular SOS cannot be an artifact of H_2_S oxidation within the extracellular buffer. Thus, the oxidized sulfur species must be generated intracellularly and effluxed out of the cells.

Table 5 compares the total small S-species concentrations, [S], under normoxic and hypoxic conditions. Hypoxia significantly lowers the overall concentration of intracellular analytes but slightly increases the rate of efflux. As seen in previous report, the effluxed [S] appears to be maintained even with the loss of intramolecular [S]. Table 6 gives the normalized analyte ratios, which demonstrate large increases of intracellular and extracellular HSOH, while the overall sulfomic indexes are only slightly affected.

### 2.4. Hypoxia-Induced Changes in Mitochondrial Function and ROS production

To understand the changes in S-speciation seen, we interrogated mitochondrial function under analogous conditions. The HAOSMCs were cultured in a HypoxyLab™ (Oxford Opronix Ltd. Milton Park, Abingdon, UK). bench-top hypoxia workstation for 48 h, followed by measurement of live-cell oxygen consumption and ROS production by high-resolution respirometry. In aerobic respiration, electrons flow through the electron transport chain via redox reactions, creating a proton gradient that drives the synthesis of ATP. Electron flow terminates with molecular oxygen being the final electron acceptor; thus, oxygen is consumed during this process. Complex I (NADH coenzyme Q reductase) accepts electrons from the electron carrier nicotinamide adenine dinucleotide (NADH) and passes them to coenzyme Q. Coenzyme Q also receives electrons from succinate dehydrogenase (complex II). Electrons are than passed from coenzyme Q to complex III (cytochrome bc1), followed by transfer to cytochrome c. Finally, electrons are passed to cytochrome c oxidase (complex IV), which transfers the electrons to molecular oxygen. The electron transfer is coupled to transfer of protons, which creates an electrochemical proton gradient that drives ATP synthesis by ATP synthase. In respirometry experiments, state 2 is defined as basal respiration, and state 3 is defined as ADP-stimulated respiration, as respiration can be accelerated in the presence of ADP via ADP-binding to ATP synthase.

**Figure 4:**
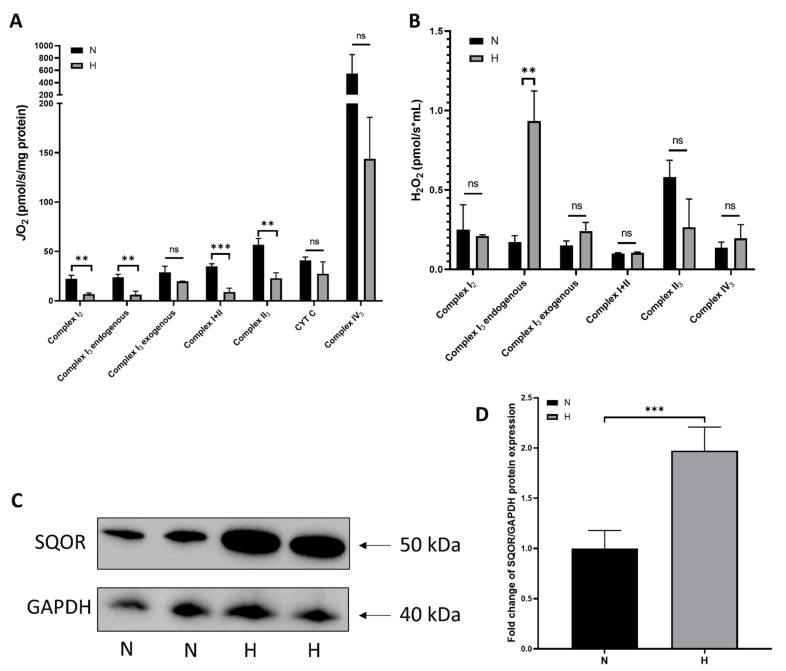
(**A**) Oxygen consumption rate (*J*O_2_). (**B**) H_2_O_2_ production rate in human aortic smooth muscle cells during normoxic (N) and hypoxic (H) growth. (**C**) Western blot showing protein expression of cellular SQOR with GAPDH used as loading control. (**D**) Fold change of SQOR protein expression for normoxia versus hypoxia. ** = *p* < 0.01; *** = *p* < 0.001; ns = not significant.

Figure 4 shows that hypoxia led to statistically relevant reduced oxygen flux (*J*O_2_), a direct measure of oxygen consumption rate during several measurement states using a substrate-uncoupler-inhibitor (SUIT) protocol: complex I, state 2 (addition of malate and glutamate); complex I, state 3 endogenous (ADP-activated); combined complex I and II (addition of succinate); and complex II, state 3 (rotenone-induced inhibition of complex I). Notably, there were no statistically relevant differences for complex I, state 3 exogenous respiration (following digitonin permeabilization of plasma membranes) or complex IV, state 3 (antimycin A-induced inhibition of complex III and addition of ascorbate/TMPD). There was no significant difference in respiration following cytochrome c addition, suggesting no difference in outer mitochondrial membrane integrity. ROS was simultaneously measured using the fluoroprobe AmR, with the addition of HRP to catalyze the reaction yielding the fluorescent resorufin, and SOD to convert superoxide to peroxide. The only statistically significant change was a large increase seen during complex I, state 3 endogenous respiration.

**Figure 5:**
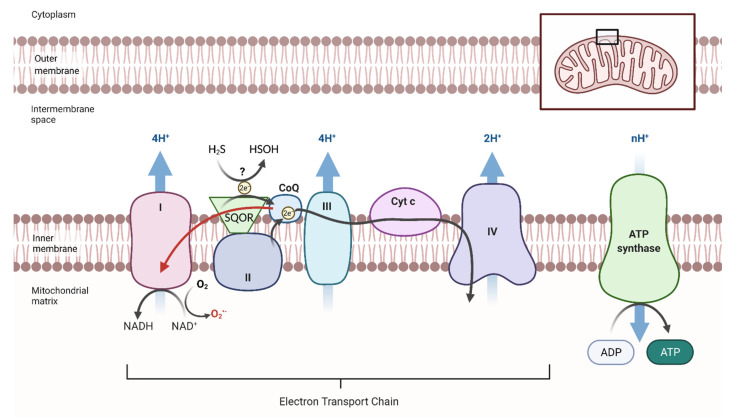
A representation of the normal mitochondrial ETC (black lines) and possible mechanism of H_2_S/SQOR-mediated reverse electron transport (red line). Under normal conditions in the electron transport chain, electrons flow through a chain of proteins that are coupled to pumping of hydrogen ions (H^+^) from the mitochondrial matrix to the intermembrane space, creating a proton gradient. ATP synthase uses the generated gradient to form ATP. Complex I accepts electrons from NADH, which are then passed to coenzyme Q (CoQ). Complex II accepts electrons from succinate, which are then passed to CoQ, similar to complex I. CoQ then functions as an electron carrier, and electrons are subsequently transferred to complex III, cytochrome c (Cyt c), and complex IV. More recently, sulfide quinone oxidoreductase (SQOR) has been identified as in integral membrane protein that interacts with the inner mitochondrial membrane. SQOR catalyzes the oxidation of H_2_S using CoQ as an electron acceptor. Under hypoxia, SQOR up-regulation may drive elevated H_2_S oxidation, overwhelming CoQ with electrons. A highly reduced CoQ pool can drive electrons backward through complex I, which increases superoxide production, ultimately measured as increased ROS.

These data suggest hypoxia induces a backflow of electrons to complex 1, a process described as reverse electron transport (RET) [91]. Western blot data shown in Figure 4C,D show SQOR expression in HAOSMC grown in hypoxic conditions was elevated 1.97 ± 0.24-fold (*p* < 0.001) compared to HAOSMC grown in normoxic conditions. In this scenario, shown in Figure 5, the loss of reduced CoQ due to diminished electron flow through complex 1 causes increased consumption of H_2_S by increased expression of SQOR, leading to increased levels of HSOH, the initial SOS metabolite of H_2_S oxidation. The respiration complexes beyond complex I are not significantly affected, but in the absence of normoxic O_2_ levels, the reduced CoQ generated by SQOR induces RET back flow to complex 1, which is a major source of ROS generation and may also lead to H_2_S oxidation. The increase in SQOR activity under hypoxia has been previously described; however, in our case, it is associated with an overall loss of trappable intracellular S-species but a substantial increase in HSOH. It is also of note that the efflux of small S-species under hypoxia increases slightly but the ratio of effluxed HSOH increases by over fourfold.

## 3. Conclusions

One important result of this investigation is the quantification of small labile S species in primary vascular cell lines. The production of H_2_S is orders of magnitude higher in smooth muscle (nanomolar) as compared to endothelial cell lines (picomolar). The distributions of the three analytes, HOSOH > H_2_S > HSOH, demonstrated much higher S-oxidation levels than seen in non-vascular cell lines [54]. Overall, the smooth muscle cells were found to have almost 25-fold higher concentrations of the endogenous S-analytes as compared to endothelial cells.

All the vascular cell lines were found to extrude the analytes extracellularly against a concentration gradient, up to 10 times higher than intracellular over analogous trapping periods. It is often suggested that H_2_S diffuses through the extracellular membrane, but at pH 7.4, the large majority of sulfide is ionized as HS^−^ or S^2−^ [39], these ions should not diffuse through the cell membrane at the rates observed. Many reports implicate H_2_S as a paracrine signaling agent at an intracellular level (i.e., cysteine persulfidation) as well as extracellular level (extracellular matrix maintenance and remodeling) [92,93,94]. However, no intramembrane channel responsible for transporting ionized H_2_S has been identified yet.

An alternative interpretation of these results is that the trapped analytes we observed are due to hydrolysis or cleavage of persulfides. Our use of the S-H-specific HPE-IAM was intended to minimize such reactivity, but the S-O specific trap dimedone has also been implicated in the cleavage of dialkylated persulfides [43]. The experimental design used, with separate traps added for each species, should also minimize such cleavage. The interconversion of persulfide and persulfide oxides has also been suggested, as well as assertions that the addition of traps changes the equilibrium between species. In this work, a consistent method has been used throughout, but the observed S-speciation changes by cell lines and conditions. Thus, we conclude that the vascular cells studied have labile S pool at higher oxidation levels than other cell lines and that small S-species are extruded in comparatively high concentrations.

Under hypoxia, smooth muscle cells showed significantly reduced oxygen consumption overall, and ROS production was found to increase during ADP-activated complex I respiration, which we suggest is due to increased SQOR activity causing reverse electron transfer, RET. A study of the endothelial cells exposed to hypoxia showed a similar reduction in oxygen consumption rate and increase in ROS production during complex I respiration [95]. Significant increases of HSOH are seen under hypoxia, despite lower overall S-species concentrations. The shift of sulfur speciation with a concomitant reduction in mitochondrial respiration emphasizes the role of H_2_S oxidation in mitochondrial function under hypoxia. Oxidized S-species display higher reactivities than H_2_S, both as reductants [55] and sulfonylating agents [49]; thus, these species may be important intermediates in processes canonically attributed to H_2_S. Distinguishing which small molecules regulate vascular functions both intracellularly and extracellularly may help clarify the paradoxical effect of a small, simple molecule such as H_2_S having biological specificity and control.

## 4. Materials and Methods

### 4.1. Materials

Reagents such as β-(4-hydroxyphenyl)ethyl iodoacetamide (CAS # 53527-07-4), dimedone (A10140-14), Trypsin^®^ (25200056-100ML), 5-sulfosalicylic acid dihydrate (247006-100G), and Hanks′ balanced salt solution (HBSS) were purchased from Sigma-Aldrich (St. Louis, MO, USA). Human coronary smooth muscle (ACBRI 443) and human coronary endothelial cells (ACBRI 377) were purchased and cultured using an optimized all-in-one-kit (Cell Systems, Kirkland WA, USA). Human aortic endothelial cells were purchased from iXCells Biotech (San Diego, CA, USA) (10HU-020, Mixed Donors), and human aortic smooth muscle cells were purchased from Gelantis (San Diego, CA, USA) (PH35405A, Donor Age: 33, Donor Sex: Male). For human aortic endothelial cells, culture medium consisted of EBM-2 Endothelial Cell Growth Basal Medium-2 (Lonza Group AG, Basel, Switzerland) supplemented with EGM-2 Endothelial Cell Growth Medium-2 BulletKit (Lonza Group AG, Basel, Switzerland), FBS to a final concentration of 20% (*v*/*v*), and 1× penicillin streptomycin (Pen-Strep) (HyClone Laboratories Inc., Logan, UT, USA) solution. For human aortic smooth muscle cells, culture medium consisted of smooth muscle cell growth medium 2 containing 0.05 mL/mL fetal calf serum, 0.5 ng/mL recombinant human epidermal growth factor, 2 ng/mL recombinant human basic fibroblast growth factor, and 5 μg/mL recombinant human insulin (Promo Cell, Heidelberg, Germany).

### 4.2. Cell Culture

Human coronary smooth muscle cells, human coronary endothelial cells, human aortic smooth muscle cells, and human aortic endothelial cells were thawed and cultured in T25 or T75 polystyrene culture flasks depending on experimental design. Cell cultures were passaged using Passage Reagent Group™ (Cell Systems, Kirkland, WA, USA.) for coronary cell lines and trypsin for aortic cell lines. All cell cultures were maintained (<4 passages) in a cell culture incubator (37 °C, 5% CO_2_).

### 4.3. Electrophilic and Nucleophilic Trapping Procedures

The human coronary smooth muscle cells, human coronary endothelial cells, human aortic smooth muscle cells, and human aortic endothelial cells were seeded in 12-well polystyrene plates at approximately 33% confluency allowed to become 80–90% confluent. Upon confluency, the cell culture media was removed, discarded, and replaced with both warm HBSS buffer solution; 150 mM stock solutions of either dimedone or -(4-hydroxyphenyl)ethyl iodoacetamide (or both traps) were immediately diluted into the HBSS buffer solution at 1% DMSO concentration for final trapping solution of 1.5 mM. After 45 min, the HBSS was removed and transferred to prelabeled 15 mL centrifuge tubes and centrifuged to pellet any unintentionally lifted cells. The 12-well plates were lifted using 300 μL of Trypsin^®^ for approximately 5 min at room temperature followed by 2 washes with 350 μL of cold HBSS buffer with all solutions transferred to the respective previous 15 mL centrifuge tubes. These cell solutions were then immediately stored at −80 °C until sample preparation.

### 4.4. Efflux Measurements

The efflux method used here involved measuring one well of a 12-well plate (with *n* = 3–6 replicates) of 50–80% confluent cells effluxing into new fractions of HBSS at 1 h increments for a total of 3 h as a measure of cultured cells ability to re-equilibrate their surroundings three consecutive times. The human coronary smooth muscle cells, human coronary endothelial cells, human aortic smooth muscle cells, and human aortic endothelial cells were seeded at 10–20% confluency in 12-well polystyrene plates first coated with Attachment Factor™ (Cell Systems, Kirkland, WA, USA.) for coronary lines or collagen for aortic lines. Upon appropriate confluency, the media was removed and replaced with 37 °C HBSS for efflux experiments. All HBSS fractions after one hour were immediately trapped upon being transferred to tubes at 1.5 mM dimedone, HPE-IAM, or dimedone + HPE-IAM and stored at −80 °C.

### 4.5. Cell Culture in Hypoxic Chamber

Hypoxia experiments were conducted in a HypoxyLab hypoxia bench-top incubator and workstation (Oxford Opronix Ltd. Milton Park, Abingdon, UK). The oxygen concentration was set at 8 mm Hg (1.0% O_2_). Human aortic smooth muscle cells were seeded into 12-well plates from a confluent T25 or T75 passage plate. All materials such as reagents, buffers, and pipet tips were placed in the hypoxia chamber before 12-well plates of cells to reduce any possible oxygen contamination during trapping experiments. Both hypoxia intracellular trapping as well as hypoxia efflux experiments were conducted in a manner identical to those described in Method 2.2 and Method 2.3. After trapping was completed, 12-well plates were removed from hypoxia chamber for trypsinization and −80 °C storage.

### 4.6. Sample Preparation

The preparation of frozen cell solutions for mass spectrometry generally occurred 24 h or less before analysis to reduce any possible decomposition of trapped cell solutions. A freeze-thaw method of quickly thawing the cell solutions from −80 °C to 37 °C in a water bath was used in absence of a detergent to reduce mass spectrometer contamination. Upon being warmed to 37 °C, centrifuge tubes containing cell lysates were then repeatedly sonicated using a microtip sonicator programmed for 90% amplitude 30 s on, 30 s off for a total of 60 s of sonication time. Due to the cell wall being present in yeast strains, 3 separate freeze–thaw cycles followed by 90% amplitude 30 s, 30 s off for a total of 120 s were used to ensure cell lysis. A 6× solution (9% *w*/*v*) sulfosalicylic acid (SSA) (Thermo Fisher Scientific, Waltham, MA, USA) solution was then added to every sample (1 mL of sample + 200 μL SSA) followed by a 5 min protein precipitation at room temperature. Samples were then centrifuged for 30 min at 200 g to precipitate high molecular weight cellular fragments and proteins, followed by the removal of approximately 1 mL of supernatant. These solutions were added to HPLC vials and stored at 4 °C until being analyzed using a mass spectrometer.

### 4.7. Mass Spectrometry

H_2_S, sulfenic (HSOH), and sulfoxylic (HOSOH) acids were trapped by derivatization reactions with nucleophilic and electrophilic reagents to form the products seen in Scheme 1, as previously described [54]. The respective concentrations were determined from calculating the selective ion chromatogram peak areas analyzed on a Q Exactive Focus Hybrid Quadrupole-Orbitrap Mass Spectrometer (Thermo Scientific, Madison, WI, USA) connected to a Thermo Scientific UltiMate 3000 Rapid Separation Quaternary high performance liquid chromatography (HPLC) system (Thermo Scientific, Madison, WI, USA). As these peak areas give a signal proportional to the number of a species (characterized by *m*/*z*) that is dissolved in a solution, a calibration curve of HPE-S-HPE was prepared from Na_2_S 9H_2_O and HPE-IAM and serially diluted to generate H_2_S solutions ranging from 1 μM to 1 nM and all unknown experimental concentrations for H_2_S, HSOH, and HOSOH were calculated using this calibration curve. The samples were injected (10 μL) into an LC system column of 100 mm × 2.1 mm dimensions with a particle size of 3 μM and a pore size (130 A) BDS HYPERSIL C18 column (Thermo Scientific, Madison, WI, USA). A binary mobile phase gradient containing 0.1% (*v*/*v*) formic acid in water (A) and acetonitrile (B) was used as the eluting solvent for a multistep gradient: 100% A for the first 4 min, continued until 15 min, then to 50% B, held for 1 min, increased to 98% B for 2 min, and then equilibrated for 4 min at 100% A. Additional chromatographic parameters were as follows: column temperature, 30 °C; flow rate, 350 μL/min. Full-scan accurate mass spectra (*m*/*z* range: 50–1200) of eluting compounds were obtained at a high resolution (70,000 fwhm) on the Orbitrap mass analyzer using an internal calibration locking against diisooctyl phthalate (plasticizer, *m*/*z* = 391.2843), with an accuracy of measurements of <2 ppm; all collected data were then processed using Xcalibur v.2.0.7 software. The electrospray source conditions were as follows: sheath and auxiliary gas flow 50 and 5 arbitrary units (a.u.), respectively; heated capillary temperature, 300 °C; electrospray voltage, 3.55 kV for + ESI; capillary voltage 43 V for + ESI; tube lens voltage, 205 V for + ESI.

### 4.8. Mitochondrial Respiration and ROS Productions

High-resolution oxygen consumption was measured at 37 °C in a respiration buffer supplemented with creatine monohydrate (20 mM) using the Oroboros O2k Oxygraph (Oroboros Instruments). Live cells were suspended in 2 mL of respiration media consisting of 105 mM MES potassium salt, 30 mM potassium chloride (KCl), 10 mM potassium dihydrogen phosphate (KH_2_PO_4_), 5 mM magnesium chloride, hexahydrate (MgCl_2_·6H_2_O), and 0.5 mg/mL BSA and added to the O2k-Chamber. A substrate inhibitor titration protocol was performed, in which 2 mM malate and 10 mM glutamate were added to the chambers to measure complex I, state 2 respiration. This was followed by 4 mM ADP addition, along with 4.05 μM digitonin to permeabilize the plasma membranes, to initiate state 3 respiration. Next, 10 mM succinate was added to the chambers to stimulate electron flow through complex II as well. Rotenone (10 μM) was used to inhibit complex I, and 10 μM cytochrome c was added to test the mitochondrial membrane integrity. Finally, complex IV respiration was determined using 0.4 mM *N*,*N*,*N*’,*N*’-tetramethyl-p-phenylenediamine (TMPD) and 2 mM ascorbate to prevent TMPD auto-oxidation, as well as 5 μM antimycin A to inhibit electron flow through complex III. The respiration rate was expressed as pmol oxygen consumed per second, normalized to protein content in the O2k-Chamber (pmols/s/mg protein). Cellular hydrogen peroxide production by cells was measured using Amplex UltraRed (AmR) (Thermo Fisher Scientific, Waltham, MA, USA) with the Oxygraph-2k FluoRespirometer and the O2k-Fluo LED2-Module Fluorescence-Sensor Green O2k (Oroboros Instruments (Innsbruck, Austria) Biorupter sonicator (Diagenode SA, Seraing, Belgium)), as previously described [96]. The reaction of hydrogen peroxide and AmR, catalyzed by horseradish peroxidase (HRP), produces a red fluorescent compound resorufin, with an excitation/emission (ex/em) of 563 nm/587 nm [97]. A 10 mM stock solution of AmR was prepared by dissolving in dimethyl sulfoxide (DMSO), and 2 μL of 10 mM AmR was titrated into the O2k-Chamber, for a final concentration of 10 μM. Additionally, a stock solution with 500 U HRP/mL (Sigma-Aldrich St. Louis, MO, USA) in respiration media was prepared, and 4 μL was titrated into the 2 mL O2k-Chamber for a final concentration of 1 U/mL. Finally, 5 U/mL of superoxide dismutase (SOD) (Sigma-Aldrich, St. Louis, MO, USA) was added to the O2k-Chamber to generate hydrogen peroxide from superoxide. Hydrogen peroxide concentrations were determined as the change of emitted fluorescence intensity as hydrogen peroxide is consumed by AmR and normalized to the oxygen consumption rate. A standard curve of the slope of the AmR fluorescence intensity was created using calibration standards of a commercial solution of 3 wt.% hydrogen peroxide (Sigma-Aldrich, St. Louis MO, USA) by titrating stepwise increases of 0.1 μM hydrogen peroxide into the O2k-Chamber. ROS production was expressed as hydrogen peroxide flux per O2k-Chamber volume (pmols/s·mL). All substrates and inhibitors were purchased from Sigma-Aldrich (St. Louis, MO, USA).

### 4.9. Western Blotting

For protein extraction, cells were rinsed 3 times with cold PBS. During the last rinse, cells were scraped with a cell scraper, and the cell suspension was transferred to a tube. Cells were then centrifuged at 1500 rpm for 10 min at 4 °C, and the supernatant was aspirated. The pellet was then resuspended in ice-cold lysis buffer, consisting of 50 mM Tris-HCl at pH 7.4, 150 mM NaCl, 1% NP-40, 0.25% Na-deoxycholate, 0.1% SDS, and 1× protease inhibitor cocktail (diluted 1:100 in lysis buffer), and incubated on ice for 10 min. Following incubation, tubes were vortexed briefly and then sonicated with a Biorupter sonicator (Diagenode SA, Liège Belgium) for 5 cycles of 30 s on and 30 s off at 4 °C. Finally, the supernatant was transferred to a new tube, centrifuged at 14,000 rpm for 15 min at 4 °C, and stored at −80 °C until analysis. To measure protein concentration in samples, a Pierce BCA Protein Assay Kit (Thermo Fisher Scientific, Waltham, MA, USA) was used. First, a set of diluted BSA standards (0–2000 μg/mL) were prepared to create a standard curve. Standards and samples were then added to a 96-well microplate at a volume of 25 μL in duplicate. Following this, 200 μL of BCA reagent, consisting of bicinchoninic acid, sodium carbonate, sodium bicarbonate, sodium tartrate, and copper(II)sulfate pentahydrate, were added to each sample and standard well. The plate was left to incubate for 30 min at 37 °C, and then the absorbance was calculated at 562 nm on a Varioskan LUX Multimode Microplate Reader (Thermo Fisher Scientific, Waltham, MA, USA). A standard curve was prepared by plotting the absorbances of the BSA standards and their concentrations. The standard curve was then be used to determine the protein concentration of the samples.

For Western blotting, protein samples were mixed with 2× LDS Sample Buffer ((Bio-Rad, Hercules, CA, USA), Hercules, CA, USA) and 2-mercaptoethanol (Bio-Rad, Hercules, CA, USA) and then heated for 5 min at 95°C in a thermal mixer. Then, using 4–15% Mini-PROTEAN^®^ TGX™ Precast Protein Gels (Bio-Rad, Hercules, CA, USA), equal amounts of protein (20 μg) were separated using electrophoresis in a Mini-PROTEAN (Bio-Rad, Hercules, CA, USA) electrophoresis system connected to a PowerPac Basic power supply (Bio-Rad, Hercules, CA, USA) at a constant voltage of 200 V for 1 h. Proteins were run in 1x Tris/Glycine/SDS Running Buffer (Bio-Rad, Hercules, CA, USA). Proteins were subsequently transferred to Immobilon-P polyvinylidene difluoride (PVDF) transfer membranes (MilliporeSigma, Burlington, MA, USA) in the Mini Gel Tank using a Mini Trans-Blot Module wet transfer device filled with 1× CAPS Transfer Buffer with 20% methanol at 50 V for 1 h and 30 min at room temperature. After transfer, membranes were washed in Tris-buffered saline containing 0.1% Tween-20 (TBST) and blocked with 5% nonfat dry milk for 1 h at room temperature. Following, membranes were washed and incubated overnight at 4 °C with primary antibody against SQOR (ProteinTech, Rosemont, IL, USA) in 5% blocking solution (nonfat dry milk in TBST), diluted 1:1000 according to manufacturer’s instructions. The membranes were washed again in TBST and incubated for 1 h at room temperature with the appropriate HRP-conjugated secondary antibody. Finally, the membranes were thoroughly washed again to remove any non-bound conjugate before being visualized using Clarity ECL Substrate chemiluminescent reagent (Bio-Rad, Hercules, CA, USA). The bands were detected using the ChemiDoc MP digital imager (Bio-Rad, Hercules, CA, USA). Membranes were subsequently incubated in anti-GAPDH antibody, and protein levels were expressed as fold changes of the protein under hypoxia, adjusted to the loading control, GAPDH, relative to the control, normoxic condition. Band intensities were quantified using Image Lab (Bio-Rad, Hercules, CA, USA).

## Figures and Tables

**Table 1:** Endogenous concentrations of analytes in primary human vascular cell lines.

Cell Type	H_2_S (pM)	HSOH (pM)	HOSOH (pM)	Total [H_2_S + SOS] (pM)
HAOSMC	2940 ± 78.8	1240 ± 73.4	5270 ± 87.3	9450 ± 63.1
HCASMC	1480 ± 136	1030 ± 12.3	1846.8 ± 102	4360 ± 170
HAOEC	75.8 ± 7.37	73.4 ± 0.901	87.3 ± 5.29	237 ± 9.12
HCAEC	110 ± 5.62	79.3 ± 0.960	130 ± 9.29	319 ± 10.9

**Table 2:** Intracellular ratios and sulfomic indexes in vascular, cancer, and non-cancer cell lines.

Cell Type	H_2_S/HSOH/HOSOH Ratios	Sulfomic Index	Total [H_2_S + SOS] (pM)
HAOSMC	1:0.42:1.79	1.27	9450 ± 63.1
HCASMC	1:0.70:1.25	1.08	4360 ± 170
HAOEC	1:0.93:1.11	1.04	237 ± 9.12
HCAEC	1:0.72:1.18	1.06	319 ± 10.9
HEK293 *	1:0.28:0.34	0.70	2610 ± 700
HeLa *	1:0.11:0.11	0.27	82.9 ± 4.55
A375 *	1:0.03:0.06	0.14	2920 ± 466

* Data from reference [54].

**Table 3:** Efflux rates of analytes in primary vascular cell lines.

Cell Type	H_2_S (pM/h)	HSOH (pM/h)	HOSOH (pM/h)	Total [H_2_S + SOS] (pM/h)
HAOSMC	6810 ± 900	3500 ± 1200	3300 ± 230	13,600 ± 1520
HCASMC	4430 ± 139	2150 ± 548	8870 ± 2120	15,500 ± 2190
HAOEC	1150 ± 742	1540 ± 1090	622 ± 404	3310 ± 1380
HCAEC	398 ± 209	88.6 ± 3.99	346 ± 45.6	833 ± 214

**Table 4:** Ratios of extruded analyte concentrations and sulfomic indexes.

Cell Type	H_2_S/HSOH/HOSOH Ratios	Sulfomic Index	Total [H_2_S + SOS] (pM)
HAOSMC	1:0.51:0.48	0.74	13,600 ± 1520
HCASMC	1:0.49:2.00	1.29	15,500 ± 2190
HAOEC	1:1.34:0.54	0.84	3310 ± 1380
HCAEC	1:0.22:0.87	0.94	833 ± 214
HEK293 *	1:0.05:0.37	0.55	2610 ± 700
HeLa *	1:0.04:0.30	0.47	82.9 ± 4.55
A375 *	1:0.017:0.057	0.12	5930 ± 899

* Data from reference [54].

**Table 5:** Normalized small S-species concentrations of analytes under normoxia and hypoxia.

Growth Condition	Total S-Species [H_2_S+SOS]	
	Intracellular (pM)	Extracellular (pM)/h
Normoxia	9450 ± 63.1	13,540 ± 301
Hypoxia	5161 ± 247	14,770 ± 371

**Table 6:** Normalized ratios and sulfomic indexes of analytes under normoxia and hypoxia.

Growth Condition	H_2_S/HSOH/HOSOH Ratios		Sulfomic Index	
	Intracellular	Efflux	Intracellular	Efflux
Normoxia	1:0.42:1.79	1:0.51:0.48	1.27	0.74
Hypoxia	1:1.20:1.68	1:2.38:0.79	1.18	0.94

## Data Availability

Additional data files are available upon request.

## Supplementary Materials

The following are available online: Figure S1: SIC of H_2_S (HPE-S-HPE) in HAOSMC; Figure S2: H_2_S calibration curve; Figure S3: Dilution factor calculations; Figure S4: Peak area to concentration calculation; Figure S5: Time-dependent efflux of H_2_S, HSOH, and HOSOH in human aortic smooth muscle cells and human aortic endothelial cells; Figure S6: Time dependent efflux of H_2_S, HSOH, and HOSOH in human coronary smooth muscle cells and human coronary endothelial cells; Figure S7: Time-dependent efflux of H_2_S, HSOH, and HOSOH in human aortic smooth muscle cells during normoxic and hypoxic growth conditions.

## Sample Availability

All cell lines used were commercially available, and all samples resulting from this work were consumed or disposed of in appropriate manner.
