# Supplementary material for: Expanding the Reactive Sulfur Metabolome: Intracellular and Efflux Measurements of Small Oxoacids of Sulfur (SOS) and H2S in Human Primary Vascular Cell Culture
Source: Molecules. 2021 Nov 26;26(23):7160. doi: 10.3390/molecules26237160 (PMC8659008; doi:10.3390/molecules26237160)
Supplement: Supplementary file 1 [file molecules-26-07160-s001.zip › molecules-1456867-supplementary.pdf]

Expanding the Reactive Sulfur Metabolome: Intracellular and Efflux Measurements of Small Oxoacids of Sulfur (SOS) and H_2S in Human Primary Vascular Cell Culture

Ottis Scrivner[1], Ahmed Ismaeel[2], Murugaeson R. Kumar[1], Kristina Sorokolet[1], Panagiotis Koutakis[2], Patrick J. Farmer[1*]

[1] Department of Chemistry and Biochemistry, Baylor University, Waco, TX 76798; Patrick_Farmer@Baylor.edu
[2] Department of Biology, Baylor University, Waco, TX 76798; Panagiotis_Koutakis@baylor.edu
* Correspondence: Patrick_Farmer@Baylor.edu

Supplemental Data

Figure S1. SIC of H_2S (HPE-S-HPE) in HAOSMC

Figure S2. H_2S calibration curve

Figure S3. Dilution factor calculations

Figure S4. Peak area to concentration calculation

Figure S5. Time dependent efflux of H_2S, HSOH, and HOSOH in human aorticsmooth muscle cells and human aortic endothelial cells.

Figure S6. Time dependent efflux of H_2S, HSOH, and HOSOH in human coronary smooth muscle cells and human coronary endothelial cells.

Figure S7. Time dependent efflux of H_2S, HSOH, and HOSOH in human aorticsmooth muscle cells during normoxic and hypoxic growth conditions

[1] Current address: Penrose TherapeuTx

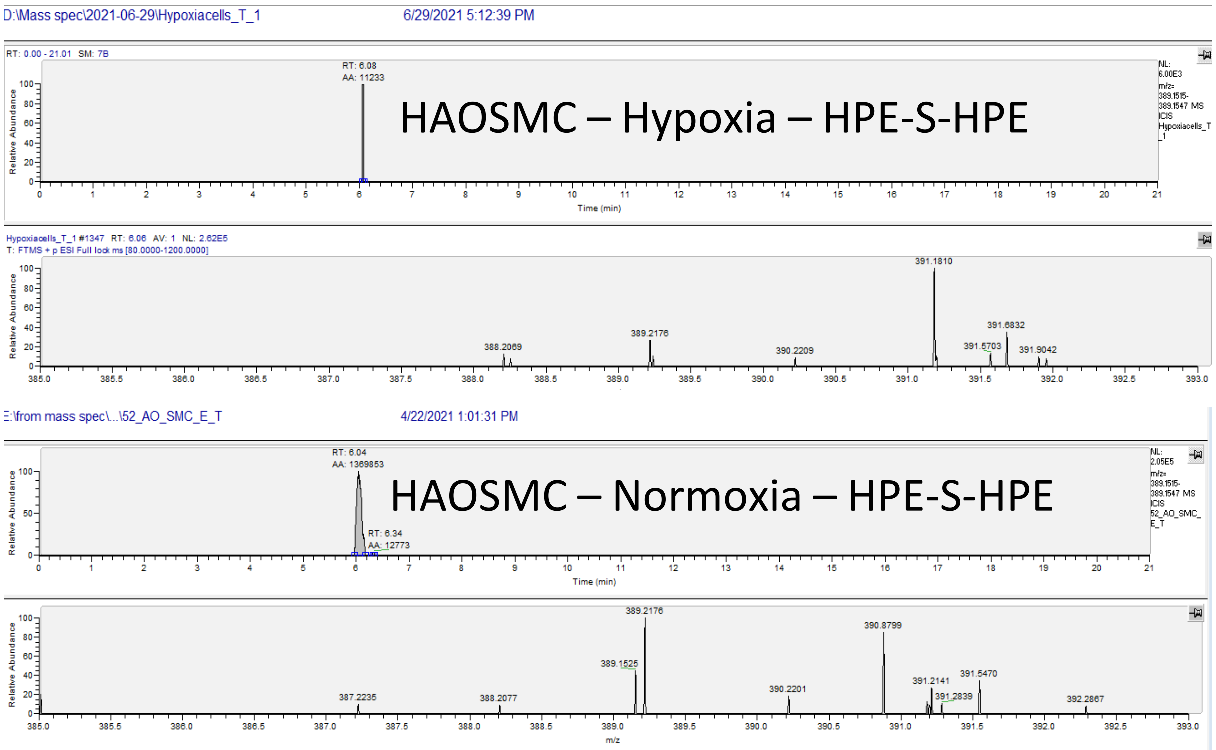

Figure S1. . SIC of H_2S (HPE-S-HPE) in HAOSMC. Selected ion chromatograms (SICs) showing the peak area of HPE-S-HPE (H_2S) forhuman aortic smooth muscle cells (HAOSMC) in both hypoxic and normoxic conditions.

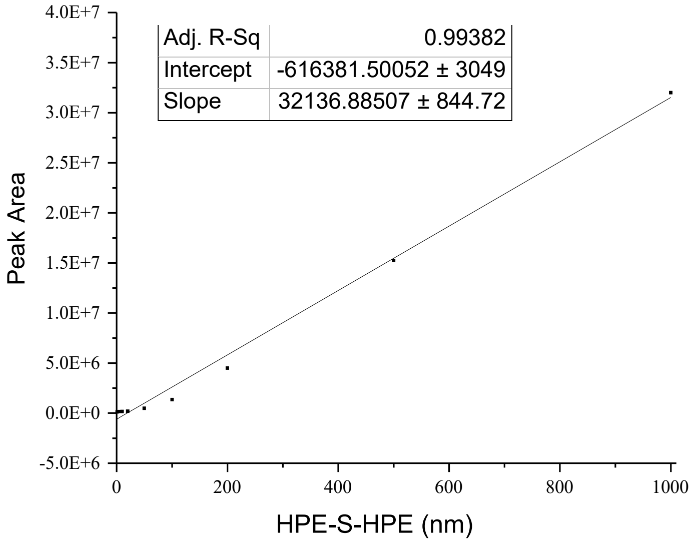

Figure S2. H_2S Calibration Curve. Calibration curve of H_2S used to translate H_2S, HSOH, and HOSOH peaks areas from chromatograms into concentrations. 100 μM stock solution of Na_2S*$9H_2O$ solution was prepared in HBSS buffer (pH 7.4) and serially diluted in HBSS buffer (pH 7.4) to generate H_2S solutions ranging from 1000 nM to 1 nM. Upon dilution, 150 mM stock solutions of HPE-IAM were then diluted 1:100 into each H_2S solution for a final HPE-IAM concentration of 1.5 mM. These trapped solutions of HPE-S-HPE were used to generate a calibration curve shown in Figure 1. Due to current difficulties in generating HSOH or HOSOH species, this calibration curve was used to calculate HSOH and HOSOH concentrations with the assumption that the ionization efficiencies of HPE-S-D and D-S-D are similar to that of HPE-S-HPE.

Table S3. Dilution Factor Calculations

							dilution factor
Cell Type	diameter µm	volume µm3	mL / cell	#cells (example)	mL of cells	uL of cells	(final volume / volume of cells
Coronary endothelial cells	13	1150.35	1.15E-09	1.83E+05	0.00021	0.21045	9503.444999
Coronary smooth muscle cells	7	17959	1.7959E-10	2.10E+05	0.00004	0.0377139	53030.84539
Aortic endothelial cells	13	1150.35	1.15E-09	127000	0.000146	0.14605	13693.94043
Aortic smooth muscle cells	7	179.59	1.7959E-10	242000	0.000043	0.04346078	46018.5022

Table S3 shows the calculations used to determine the dilution factor needed to correct for what is in practice a very small volume of cells generating sulfur species versus the final volume of solution after sample processing is completed. Cell diameters have been determinedfrom those established in the literature as well as contacting the cell vendor for their technical expertise. Those diameters were converted into volume (μm^3) followed by a conversion to mL /cell. This volume (mL / cell) is multiplied by a number of cells determined by using a Countess® Automated Cell Counter hemocytometer with trypan blue staining to determine thedilution factor. This dilution factor can then be used to correct for any dilution in concentration (**See S4)** that occurred during sample processing.

	y = 32136x + (-616381)							
							dilution factor	18716.76896
HPE-S-HPE	Peak area (y)	b	y+b	m	conc	#cells	conc/cell	*dilution
	1.31E+05	616381.5	7.47E+05	3.21E+13	2.33E-08	357000	6.51E-14	1.22E-09
	3.06E+05	616381.5	9.22E+05	3.21E+13	2.87E-08	357000	8.04E-14	1.50E-09
	2.89E+05	616381.5	9.05E+05	3.21E+13	2.82E-08	357000	7.89E-14	1.48E-09
	3.56E+05	616381.5	9.73E+05	3.21E+13	3.03E-08	357000	8.48E-14	1.59E-09
	3.58E+05	616381.5	9.74E+05	3.21E+13	3.03E-08	357000	8.49E-14	1.59E-09
							avg	1.48E-09
							std	1.36E-10

avg	HCASMC
HPE-S-HPE	1.48E-09
std	HPE-S-HPE
HCASMC	1.36E-10

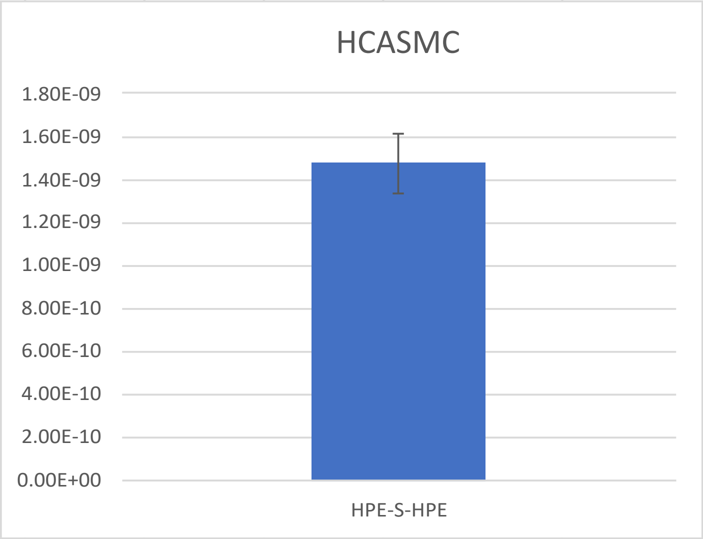

Figure S4. Example Calculation. An example of a calculation converting the peak area recorded from a SIC into concentration using the slope intercept equation determined from the calibration curve in S2. After the peak area is converted into concentration, the value is divided by the number of cells recorded for said sample and multiplied by the dilution factor determined as shown in S3.

	HAOSMC			HAOEC		
avg	HSH	HOSH	HOSOH	HSH	HOSH	HOSOH
1H	6193	4925	3478	331	314	122
2H	6725	3014	3180	1093	2622	948
3H	7398	2516	3188	2026	1836	797

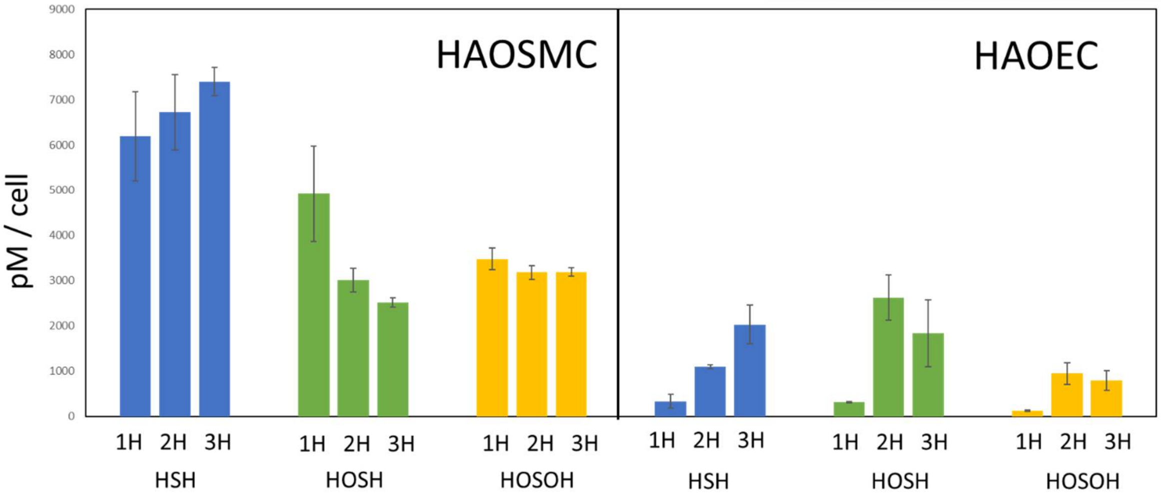

Figure S5. Time dependent efflux of H_2S, HSOH, and HOSOH in human aortic smooth muscle cells and human aortic endothelial cells. Individual hour time points of effluxed species for both human aortic smooth muscle cells (HAOSMC) and human aortic endothelial cells (HAOEC). Upon cell confluency, cell media was removed and replaced with HBSS buffer (pH 7.4) for 1, 2, and 3-hour increments. After each hour time point buffer was removed and new buffer was added to cells; the removed buffer fractions were then added to 15-mL centrifuge tubes and either HPE-IAM, HPE-IAM + DMD, or DMD only was to each tube.

		HCASMC			HCAEC	
avg	HSH	HOSH	HOSOH	HSH	HOSH	HOSOH
1H	3949	1983	7671	592	86	311
2H	4443	2747	11708	314	90	325
3H	4907	1732	7231	287	90	402

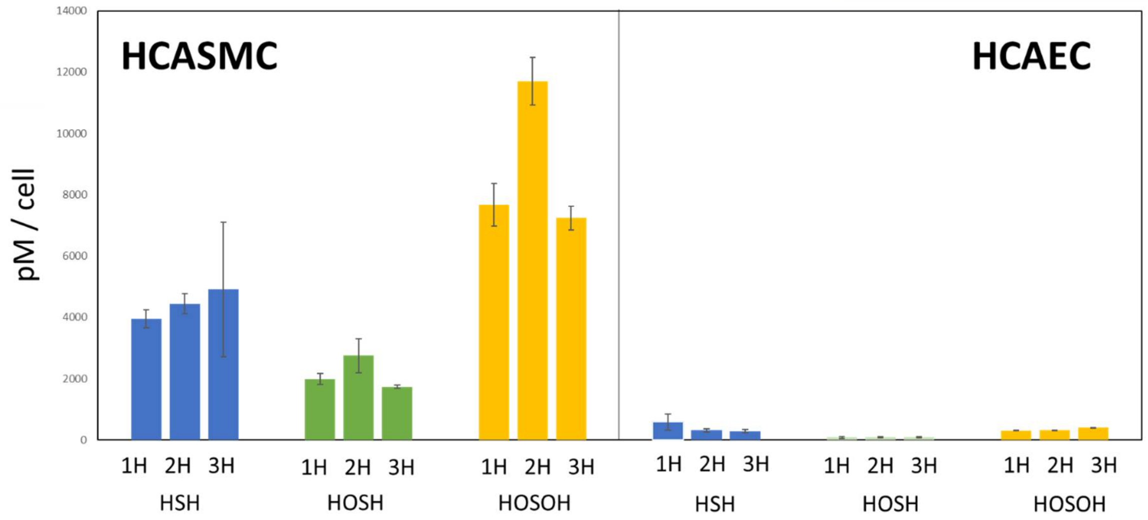

Figure S6. Time dependent efflux of H_2S, HSOH, and HOSOH in human coronary smooth muscle cells and human coronary endothelial cells. Individual hour time points of effluxed species for both human coronary smooth muscle cells (HCASMC) and human coronary endothelial cells (HCAEC). Upon cell confluency, cell media was removed and replaced with HBSS buffer (pH 7.4) for 1, 2, and 3- hour increments. After each hour time point buffer was removed and new buffer was added to cells; the removed buffer fractions were then added to 15-mL centrifuge tubes and either HPE- IAM, HPE-IAM + DMD, or DMD only was to each tube.

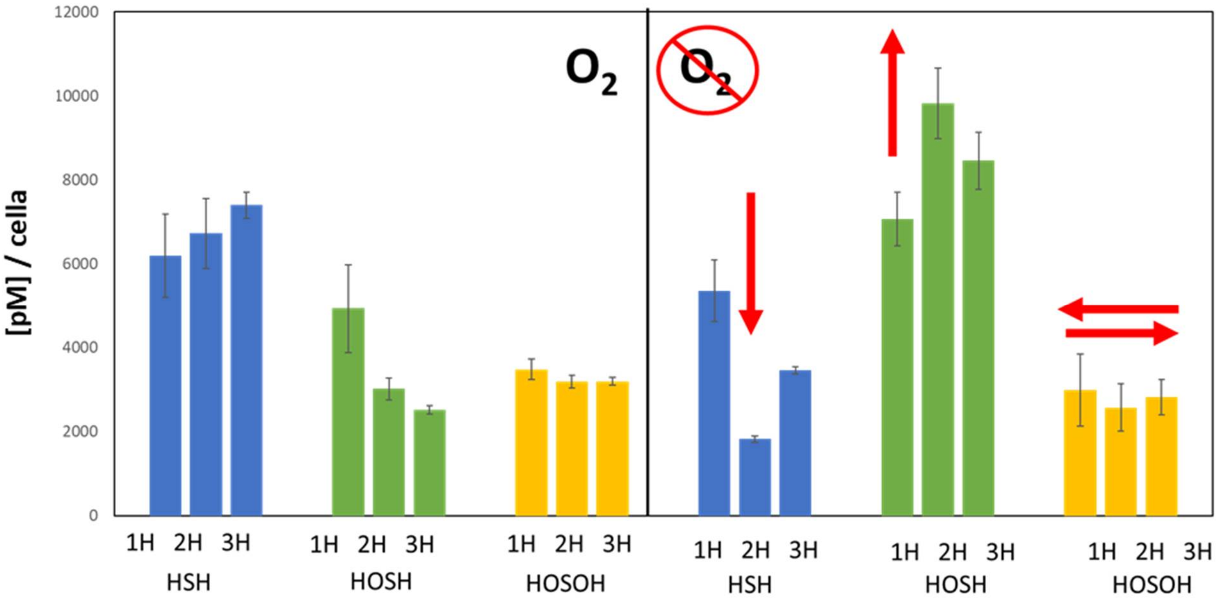

Figure S7. Time dependent efflux of H_2S, HSOH, and HOSOH in human aortic smooth muscle cells during normoxic and hypoxic growth conditions
